# Assessing a Digital Scorecard on Global Immunization Progress: Stakeholder Views and Implications for Enhancing Performance and Accountability

**DOI:** 10.3390/vaccines12020193

**Published:** 2024-02-13

**Authors:** Rose Weeks, Padmini Vishwanath, Katy Atkins Stewart, Christine Liang, Oniovo Efe-Aluta, Folake Olayinka, Carolyn Inae Kim, Erlyn Macarayan, Lori Niehaus, Naor Bar-Zeev, Chizoba Wonodi

**Affiliations:** 1 United States Agency for International Development (USAID) MOMENTUM Country and Global Leadership, Baltimore, MD 21231, USAkaty.stewart@aspeninstitute.org (K.A.S.); cwonodi1@jhu.edu (C.W.); 2 Department of International Health, Johns Hopkins Bloomberg School of Public Health, Baltimore, MD 21231, USA; 3 World Health Organization Regional Office for Africa, Brazzaville P.O. Box 06, Democratic Republic of the Congo; efeo@who.int; 4 Public Health Institute, USAID Global Health Training, Advisory and Support Contract Project, Washington, DC 20045, USA; folayinka@usaid.gov; 5 World Health Organization, 1211 Geneva, Switzerland; kimin@who.int (C.I.K.); macarayane@who.int (E.M.); barzeevn@who.int (N.B.-Z.); 6 Centers for Disease Control and Prevention, Atlanta, GA 30329-4027, USA; tvf1@cdc.gov

**Keywords:** immunization, data visualization, population health, implementation research, usability, evaluation, formative research

## Abstract

Global health agencies and regional and national stakeholders collaborated to develop the Immunization Agenda 2030 Scorecard, a digital data visualization platform displaying global, regional, and country-level immunization progress. The scorecard serves to focus attention and enable strategic actions around the measures visualized. To assess the scorecard’s usability, appropriateness, and context for use, we interviewed 15 immunization officers working across five global regions. To further understand the implementation context, we also reviewed the characteristics of 15 public platforms visualizing population health data. We integrated thematic findings across both methods. Many platforms highlight service gaps and enable comparisons between geographies to foster political pressure for service improvements. We observed heterogeneity regarding the platforms’ focus areas and participants’ leading concerns, which were management capacity and resourcing. Furthermore, one-third of platforms were out of date. Results yielded recommendations for the scorecard, which participants felt was well suited to focus the attention of decision makers on key immunization data. A simpler design coupled with implementation strategies that more actively engage policymakers would better align the scorecard with other public platforms engaging intended users. For population health platforms to serve as effective accountability mechanisms, studying implementation determinants, including usability testing, is vital to meet stakeholder needs.

## 1. Introduction

Vaccines are one of the world’s most powerful public health interventions, preventing up to five million deaths each year from deadly diseases and enabling children to thrive into adulthood [1]. Immunization strategy increasing takes a life-course approach with vaccination extending from childhood into adulthood [2]. Scaling up coverage of recommended vaccines, including against pneumococcus, rotavirus, and HPV, to 90% globally by 2030 would avert 50 million deaths [3]. Newer vaccines increasingly protect adults across the lifespan, with COVID-19 vaccines estimated to have saved the lives of over 20 million people in the first year following the introduction of vaccination [4]. Yet even before the COVID-19 pandemic caused significant backsliding in vaccination coverage, with rates decreasing in 2021 to the lowest level in decades [5], access to routine immunization services had stagnated, with progress threatened by increasing rates of conflict, migration, climate change, urbanization, and other sources of instability [6].

Global health agencies and regional and country stakeholders collaborated to develop the Immunization Agenda 2030 (IA2030) Scorecard, a digital data visualization platform showcasing performance across 18 immunization indicators at the country, region, and global level available at https://scorecard.immunizationagenda2030.org/ (accessed on May 2022) [7]. The scorecard summarizes indicators tracked under the Immunization Agenda 2030: A Global Strategy to Leave No One Behind, endorsed by the 73rd World Health Assembly in WHA73/(9). IA2030 is a global strategy requiring broad ownership by stakeholders, including those addressing health systems strengthening and disease-specific initiatives (e.g., measles and polio) [8]. IA2030′s monitoring and evaluation strategy, detailed in its Framework for Action, emphasized the need for “rebuilding of immunization programmes” after the cataclysm of the pandemic subsided [8].

Launched in 2022, the IA2030 Scorecard aims to monitor progress and increase shared accountability by partners at all levels, including national and regional immunization technical advisory groups, country-led mechanisms, and global agencies and funders [8]. Accountability is described in the Framework as supporting stakeholders to commit to goals, justify actions, implement data monitoring, and “make effective, efficient, and equitable progress toward agreed … goals” [8]. Updated annually, the scorecard—so dubbed because of its mission to enable strategic decision making around long-term goals—is one of several ownership and accountability tools displaying actionable data for immunization leaders and advocates. It serves to focus attention and enable strategic decision making around the Framework’s global indicators, seven Impact Goals and 15 Strategic Priorities, which encompass a range of measures from health worker availability to subnational vaccine coverage [9].

Health systems have used scorecards for performance management for several decades. Business management theorists Kaplan and Norton designed the balanced scorecard tool in 1992 to combine measures from diverse perspectives, including customers, finances, business, and innovation and learning, emphasizing teamwork, a long-term management perspective, and “fostering a culture of accountability” [10,11]. Healthcare organizations subsequently adopted variations of this tool to track patient satisfaction as well as financial performance [12]. Noting the successful integration of scorecards in Dutch and Italian public health care systems, in 2004, Afghanistan successfully adopted a balanced scorecard to improve health service capacity and service delivery [13]. Meanwhile, as the volume of available data grows across healthcare, performance dashboards are increasingly used to provide interactive and iterative performance readouts, positioned as “real time” reports [14,15]. Adopting “easy to read” performance data in scorecards and dashboards, such as line and bar chart styles, is used to increase stakeholders’ awareness of performance deficiencies and ensure providers know what outcomes to prioritize [11]. The concurrent growth of the visual analytics field, in which large amounts of data are displayed in graphical formats to help users with diverse backgrounds better understand data, communicate results, and disseminate information, meant there was a range of technical and stylistic options to visualize IA2030 data [16].

Stakeholder engagement is a critical factor in the implementation of all such tools, and visualized data must be accessible and meaningful to intended users [17]. Yet limited evidence is available in the peer-reviewed literature about how platforms have surveyed users to assess usability—how effective, efficient, and satisfying the platforms are in helping users complete tasks [18]. As such, we sought to gain perspectives on the understandability and appropriateness, or perceived fit, of the scorecard for intended users [19]. This was performed through a series of key informant interviews (KIIs) with globally representative immunization officers working for governments, UN agencies, and allied organizations. Our study was guided by the following research questions:

1. What are the informational needs of immunization program officers related to monitoring performance and communicating about immunization systems?
2. How do stakeholders see the appropriateness of the IA2030 scorecard, and what are its use cases?
3. What design changes would better enable stakeholders to enhance accountability, track program performance, and advocate for resources?
4. How have other health initiatives used digital visual analytic platforms (i.e., dashboards and scorecards) to enhance accountability, track program performance, and advocate for resources?

To answer the last research question, we used a document review strategy to catalog existing dashboards and scorecards in population health, seeking to understand the design and engagement strategies deployed across the field. Finally, we aimed to understand whether the informational needs and expectations for the IA2030 scorecard articulated by regional and country-level stakeholders align with dashboards and scorecards created to monitor other public health issues/movements, or whether participants’ expectations around the IA2030 scorecard were unique.

## 2. Materials and Methods

This paper integrates findings from a key informant interview series with immunization managers and a review of other publicly available visual analytic platforms developed for health advocacy and accountability.

### 2.1. Key Informant Interviews

Once the research questions were defined, the research team identified a research method, sample size, participants, and analysis plan. Qualitative key informant interviews were selected as the most appropriate method to understand end users’ individual informational needs, their experiences with scorecards, and gain detailed feedback from stakeholders. Based on our target user for the IA2030 scorecard, we sought to interview English-speaking regional and country-level technical routine immunization officers and program managers representing themselves in their professional capacities. We used a purposeful and snowballing sampling strategy to identify interview participants in positions of responsibility that made them conversant in immunization management and policy priorities. We identified participants through leadership at the World Health Organization’s (WHO) Immunization, Vaccines, and Biologicals department, the Sabin Vaccine Institute’s Boost community, and the Geneva Learning Foundation’s Movement for Immunization Agenda 2030; the latter two networks had hosted listening sessions on the scorecard’s initial designs [20]. After identifying participants working at the regional level through WHO, we used the roster of listening session participants to identify participants, attempting to complete interviews with participants representing all WHO regions, varying levels of decision making, and a mix of agency, governmental, and nonprofit organizational affiliations, prioritizing interviews with participants working within health ministries. We also used snowballing methods, in which potential respondents we initially contacted referred us to colleagues with more knowledge of the IA2030. Once data saturation was reached, as measured by opinion range, complexity, resonance with existing literature, and expert-validated findings, we stopped recruitment [21].

#### 2.1.1. Recruitment and Interview Process

Coinvestigators sent an invitation email to selected study participants with the objectives of the interview and a link to an initial version of the IA2030 scorecard, featuring only the Impact Goals, which launched in May 2022. Then using a nonpublic development website, interviewers displayed additional scorecard pages slated to launch in October 2022 during interviews, demonstrating the functionality of the indicator pages, country pages, and overview dashboard, which has simplified visualizations of all 18 indicators with a visual icon, a blue check mark or red exclamation mark, showing if each Impact Goal is on-track to reach 2030 targets and whether each Strategic Priority indicator has improved since the previous reporting year. Four of the 22 Strategic Priority indicator pages had not yet been developed. Participants were advised during the consent process that they were free not to respond to any question and could withdraw from the study at any time. All efforts were made to protect the confidentiality and privacy of study participants throughout the study.

Interviews were conducted and recorded over Zoom, transcribed using Temi, and cleaned by two team members. One interview was initiated in English and completed partially in French, with the transcription completed manually.

#### 2.1.2. Analysis

Based on the cleaned transcriptions, investigators used a modified framework analysis method to conduct inductive thematic analysis [22,23]. The data were analyzed for overarching themes by one team member. These initially identified themes were then discussed among three research team members and further refined based on these discussions. All three team members then coded a subset of interviews, and some themes were either discarded or merged with others based on their findings. With a finalized codebook, team members coded the remaining interview transcripts using Atlas 22.2.0.

Furthermore, thinking from an implementation science lens, we grouped codes into three of the five domains described by the Consolidated Framework for Implementation Research (CFIR) metaframework [24]. The *outer setting* represented the political and social context within which an organization resides, while the *inner setting* included organizational-specific or other contextual facets that will affect scorecard use. *Product characteristics* were analogous to CFIR’s intervention characteristics.

Investigators created one chart per interview (template in Appendix A) with rows for each code, organized by CFIR-related category, and associated interview excerpts placed in columns. Team members developed a summary note per code. Summary notes from each code of each chart were used to build a summary table, with the table enabling comparisons between respondents. Finally, data were condensed to highlight core findings for each code as well as convergence or divergence between country-level and regional respondents.

### 2.2. Document Review

We focused our document review on publicly available web platforms that displayed data visually and pertained to health system management, particularly tools described as supporting advocacy and accountability for system improvements. After an exploratory search using PubMed for platforms that are or were publicly available (sample terms used: MeSH:metric; scorecard or dashboard, NOT MeSH:social media), we used Google (terms included: balanced scorecard, immunization scorecard/dashboard, vaccine scorecard/dashboard, population health scorecard/dashboard, and public health scorecard/dashboard) and snowball sampling via coinvestigators to identify platforms that were publicly available, had been updated at least once, and showed evidence of use. To document evidence of use by influential stakeholders, we included platforms that listed media articles citing their data or, alternatively, searched news archives to understand if the platforms’ data were cited. We also included platforms describing policymaker-focused engagement, for instance, elected leaders citing the data in public testimony. As many such dashboards were launched from 2020 to 2022 to monitor the spread of and response to COVID-19, we included a smaller number of these tools that were emblematic of this dashboard type and had evidence of having influenced public awareness and accountability. Furthermore, several health agencies have launched visual analytics dashboards, and our review focused on an illustrative subset. Rather than compiling a comprehensive repository of such tools, we sought to identify a subset of platforms with evidence of having reached target users. We extracted data into charts to understand the attributes of each tool, including 21 fields such as components, mission, visual styles, purpose, and evidence of use. During the analysis, we sought to understand the evolving nature of public health analytic platforms, using the READ document review approach to attain saturation [25].

### 2.3. Integrated Qualitative Findings

A matrix, shown below, helped investigators assess convergence and divergence between document review and interview findings across six of ten core themes. Four themes were not included in the table because two themes pertained uniquely to the IA2030 scorecard—had no salience with other platforms reviewed—and two were bundled and analyzed with a similar theme.

## 3. Results

### 3.1. Key Informant Interviews

*Respondent Characteristics.* Fifteen key informants were interviewed in 2022. Four participants worked in the Americas region (i.e., AMR), one in the European region (EUR), five in the African region (AFR), four in the Eastern Mediterranean region (EMR), and one in the South-East Asia region (SEAR). Participant characteristics are summarized in Table 1. Three of the participants identified as female, and 12 identified as male. Two individuals worked at the subnational level, six worked at the national level, and seven worked at the regional level. Their roles either focused on monitoring and evaluation (M&E), day-to-day program management, or technical support. Several respondents did not respond to interview requests; these tended to be from AMR, and we presumed they preferred not to conduct interviews in English. One interview was terminated early when the interviewer recognized that the individual was not involved in the routine immunization program and did not fit the inclusion criteria.

### 3.2. Interview Transcript Analysis

**Figure 1:**
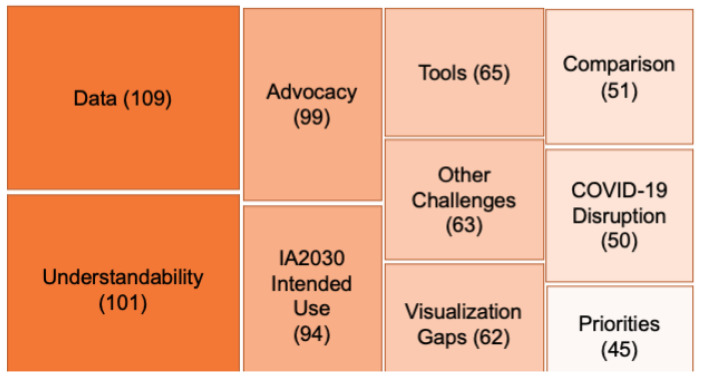
Interview themes, by frequency.

To focus our analysis on the most salient topics from the perspective of our participants, we extracted and condensed textual excerpts identified with the most frequently applied 10 codes, as seen in Figure 1, which were Data (109 coded instances), Understandability (101), Advocacy (99), IA2030 Intended Use (94), Tools (65), Other Challenges (63), Visualization Gaps (62), Comparison (51), COVID-19 disruptions (50), and Priorities (45); Appendix A lists codes and definitions. Topics are listed according to saliency, for instance, the number of times mentioned across all interviews.

#### 3.2.1. Outer Setting Context for Scorecard Use

**Figure 2:**
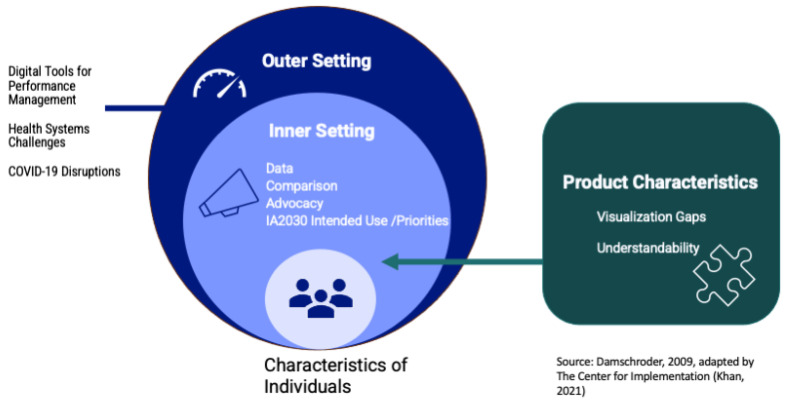
Interview themes, by CFIR construct [24].

*Digital tools for performance management.* The most common theme participants discussed under Outer Setting, as seen in Figure 2, was Tools [26]. Excerpts coded under *Tools* included a discussion of data visualization or management tools to monitor immunization systems at the country or regional level. Such tools, including their limitations, were a focus for participants discussing their information needs for monitoring and communicating about immunization systems. At the country level, participants expressed increasing familiarity with dashboards, yet some pointed to technological limitations, such as training requirements and the need for smartphones. Specifically, several country-level participants discussed using COVID-19 dashboards and DHIS2, which participants described as having a password-protected data display called a dashboard.

“*At the end, COVID-19 introduced a lot of new things in the program [like] data visualization dashboards… [and the] use of digital tools,*” said a regional monitoring specialist [KII01].

A country-level officer from the Americas, KII02, described the routine use of a COVID-19 dashboard to review progress and “*facilitate dialogue*” between teams.

“*We were having weekly meetings [to] prepare for the implementation of COVID vaccine. And that was the tool we were looking at every time we had a meeting, so we would start a meeting with this tool, and we would say, okay, that’s where we are in terms of objectives.*”

While the growth of dashboards was seen positively, regional participants described wanting guidance on how existing dashboards relate to the IA2030 scorecard. One participant, KII03, described integrating (provisional) surveillance data into the scorecard.

“*[Our] region uses a surveillance system for VPD [vaccine-preventable diseases]—polio, measles, rubella and CRS. We do a weekly data bulletin to show the data. It should work hand in hand … fit in the scorecard because the indicators [are] used in all the regions of WHO.*”

*Health system challenges.* This code, listed above as “other challenges,” captured discussions of barriers to better immunization performance, other than pandemic-related disruptions (described below); such considerations were relevant in the outer setting or implementation context for the IA2030 scorecard. Participants often discussed ongoing management and accountability-related barriers to program performance, including managerial capacity. Country-level participants contrasted donor priorities with local priorities. Regional participants stressed that a lack of financial resources limited program planning—for instance, the formation of working groups that increase coordination and accountability. Both types of participants discussed poor governance and instability as crucial challenges.

*COVID-19 Disruptions.* This theme described pandemic response, vaccine implementation, and system disruptions. Country- and regional-level respondents emphasized enormous COVID-19-related performance setbacks, stressing the diversion of funds and the pressure on staff whose workloads significantly increased. Managers often prioritized pandemic-related service delivery over routine vaccination. Country-level participants discussed the impact of social distancing policies (e.g., curfews and facility closures) and supply chain disruptions. One participant, KII03, said:

“*It was difficult to get the vaccines to [people] on time during the pandemic. There’s also the problem of personnel, there’s not a lot of people you could mobilize and the priority went to COVID vaccination.*”

Participants discussed an erosion in trust and a decline in vaccine demand. Several felt the pandemic had exacerbated existing disparities in country performance, where some countries have recovered while others have not.

#### 3.2.2. Inner Setting Context for Scorecard Use

Several inner setting factors represent structural and intra-organizational political contexts that will affect how users may adopt the scorecard for monitoring at the regional and country levels.

*Data.* This code described discussions of data units and standardization. Participants most often discussed data quality and timeliness issues. Regional participants were more likely to observe discrepancies in the scorecard data, for instance, between administrative data reported by countries through electronic Joint Reporting Form (eJRF) and sometimes divergent WUENIC data. Some country-level participants using national dashboards like DHIS2 worried about potential discrepancies.

Most country-level participants suggested including subnational data, which could be updated frequently. One, KII05, said the scorecard could then “*mobilize local resources*” to strengthen the program at those levels, continuing:

“*If we had the data further broken down to regional or district level, it becomes useful also at the subnational level for managers of the EPI at the regional and district level for purposes of advocacy.*”

Both participant types felt baseline measures and sources were insufficiently cited on the scorecard Overview and Country pages. Regional participants emphasized that data must be up-to-date to be credible for policymakers.

“*If it’s not timely, then it’s not going to be reliable, and then it becomes a library,*” said a regional manager [KII10]. “*By the time you are publishing the book, it’s already outdated.*”

Regarding data comprehensiveness, regional participants said the scorecard should include mixed data types, such as qualitative data, and broader geographic inclusion of territories.

*Advocacy.* These coded segments were used where participants discussed the use of the IA2030 scorecard for decision making and implementation, for instance, using scorecard visuals in advocacy presentations or other high-level communications to policymakers. Participants said the scorecard could be used for briefing decision makers, rapidly identifying program gaps, allocating resources, and advocating for policies. One country-level immunization manager, KII05, said the country page would be “*extremely useful as part of engagements with partners on where our weaknesses are.*”

Another country-level manager, KII11, described the use of the scorecard in meetings as a credible data source to inform decision makers:

“*They need to understand the data. It is good for us to have [data] here in scorecard so that we don’t have any doubt.*”

Both participant types discussed using the scorecard to engage Ministry of Health actors and civil society organizations, such as NGOs, in data-driven policy discussions, noting the tool’s ability to quickly convey high-level visual information about key programmatic issues to inform decision making. Both also discussed the relevance of the scorecard to subnational actors. A technical officer, KII09, said there should be a designated engagement channel with ministry officials:

“*There should be a way of communication [in] negotiation with the ministry of health regarding their data here [on the scorecard].*”

Regarding the overview dashboard, one regional monitoring expert [KII04] assessed its design: “*It’s just having a quick visual of which goals are on track and which are not*.”

Country-level participants said the scorecard will facilitate advocacy and management functions at the national level, including ensuring that the program is on the right track, prioritizing areas shown to be off-track, and identifying coverage gaps. They also felt the scorecard would help with budget decisions, facilitate dialogue between teams by understanding each other’s data, and provide a tool for local community engagement.

Regional participants suggested the scorecard could help them engage with different actors but that the tool facilitated “*high-level*,” not “*day-to-day*,” advocacy because of its lack of “*granular detail*” at the country level. They said the scorecard will support resource allocation, for instance, campaign funding and the prioritization of technical support for countries lacking capacity.

“*When we will have a scorecard showing trends of countries over years, this will generate more power to support our requests for funding and support for countries,*” said one regional immunization manager, KII07, “*definitely it’s going to help.*”

These participants also felt the scorecard would support advocacy and government accountability for issues highlighted in the scorecard, such as new vaccine introductions.

*IA2030 Intended use and Priorities.* The code *IA2030 intended use* was applied when participants referred to the use or tailoring of IA2030 objectives and indicators for immunization strategy in their country or region (“intended” denoted the focus of country ownership in the IA2030 Framework for Action) [8]. *Priorities* referred to current or new changes in prioritization in immunization programs.

Participants both at the regional and country levels said the IA2030 movement supports country and regional priority setting by shifting the focus of decision makers from immediate needs to longer-term strategy. Both regional-level and country-level participants said the framework offers priorities to refer to when planning at the regional and district levels and that they hoped the agenda would increase political accountability and result in more targeted funding. Several country-level participants referred to the challenge of integrating IA2030 priorities with other strategies, such as Gavi 5.0, into national planning documents.

Most regional participants discussed the compatibility of regional frameworks with the current scorecard structure, commenting that the scorecard appears to be designed for use at the global level and needs to be adapted for regional and country use with tailored sets of indicators (for an example of regional tailoring, see Box 1).

“*It’s important that this tool is helpful for [the regions] not just for the WHO, UNICEF, or the donors,*” said one regional monitoring specialist, K001.

Regional participants were more likely to reflect on the challenges of the dynamic nature of the IA2030 M&E framework. See Box 1 for an example of how one region has customized the framework.

Box 1 **Regional Frameworks for Action.**

WHO regions can modify the IA2030 global Framework for Action to fit their contextual needs. Adaptation techniques are unique to every region. In the case of AFRO, a survey was conducted to prioritize Impact Goals and Strategic Priorities. Ranking survey results yielded regional priorities. The top six key focus areas include sustainable immunization in primary health care; effective leadership, governance, and management; strong supply chains and logistics of vaccine deployment; vaccine-preventable disease surveillance; addressing low coverage among disadvantaged populations, and; availability and appropriate distribution of skilled health workers.

*Comparison.* This code referred to discussions about the dashboard fostering comparisons across indicators or between countries and regions. Country-level participants said the scorecard furthers the IA2030 agenda by benchmarking country performance against global performance, regional metrics, and country peers. In the words of a program manager in a middle-income country, KII05:

“*If we’re measuring progress,… it’s more useful if we compare with our peers, countries that have similar characteristics and context while also looking at the performance against global targets.*”

Participants discussed how cross-country comparisons can highlight high-performing countries and those using resources efficiently to meet targets. Country-level participants said the potential for infectious disease spillovers made country comparisons with neighbors highly relevant. They said that comparing subnational data, if added to the scorecard, would be useful for decision making at the national level.

Most regional-level actors agreed it would be beneficial for countries to compare themselves to others in the region, putting “*positive pressure*” on program managers. A minority of respondents worried about comparisons being politically insensitive. Participants felt the scorecard was missing a feature to compare regions, which could spur action at the regional level.

#### 3.2.3. Product Characteristics

Participants discussed the scorecard’s product characteristics, including its relative advantage and design quality; product adaptability was explored above in *IA2030 intended use*. Two related themes participants discussed extensively were understandability and visualization gaps.

*Understandability.* This code, a construct associated with health literacy, referred to participants’ ease or difficulties interpreting the scorecard, including a discussion of design strengths and weaknesses [27]. This could refer to the language used, visualization, color schemes, or the meaning of “*on track*” or “*off track*.”

Most participants thought the scorecard graphics were easy to interpret. One national program manager, KII06, commented on the overview dashboard as being easy to understand:

*The graphical representation is quite attractive. It is very easy to look at what is missing, off track, on track, where we are standing.*

However, other country-level respondents identified the overview dashboard or regional comparison feature of country pages as too complex. A country-level respondent, KII03, thought the scorecard would be “*a very good resource*” but that the Overview page was too lengthy, saying “*scrolling is a problem*.”

Regional-level respondents overall felt the scorecard was acceptable and easy to use, with dynamic, appealing visuals, although the country pages were flagged as less easily interpretable.

“*If you look at 12.2 as a basic reader, I don’t know if this is fine or not fine*,” said a specialist from the Americas, KII02, referring to the numerical indicator, *health workforce density per 10,000*, shown on a country page that depicted placeholder data. “*It really is important that everything be self-explanatory*.”

Both regional and country-level participants said “on track” or “off track” status should be better defined for dashboard views and suggested ways to improve user interpretability. For instance, a technical comparison on country pages between country-level and regional measures was not easily understandable for participants. Another graphical presentation that confused some participants was a stacked bar chart display that required users to interact with a mouse hover to reveal component categories (i.e., Impact Goal 1.1). Both participant types said it would be easier to understand vaccination coverage if the chart styling was standardized; coverage was depicted variously in line and bar charts.

*Visualization gaps.* These coded excerpts described features missing from the scorecard. Few country-level participants discussed the need for additional data visualizations, although one recommended displaying data via maps instead of charts. Regional participants requested enhanced regional views, for instance, displaying the performance of member countries, country rankings, goals achieved, and comparisons between regions. Several regional participants, discussing the range of country performance, suggested highlighting differences between countries by their income status.

Some participants wanted to view available resources (i.e., financial) related to achieving immunization goals. Regional participants shared ideas for enriching the country pages, for instance, by depicting data trends over time.

### 3.3. Document Review

We identified a representative sample of 15 publicly available visual analytics resources developed to increase public awareness and accountability for population health measures (full list in Appendix B). The platforms described their purpose as public monitoring tools to track performance, “understand our progress,” and identify “health systems gaps” (Goalkeepers, ALMA). Several sought to provide “actionable data,” stating that “the use of data drives better results” (Congressional, UNICEF). Two explicitly sought to “improve accountability” (ALMA Scorecard for Accountability and Action, Vermont’s Immunization & Infectious Disease Scorecard), or stated that they intended to “help… policymakers… respond” to the COVID-19 crisis (Johns Hopkins Coronavirus Resource Center).

Tools use established frameworks, such as the U.S. Healthy People initiative, Sustainable Development Goals, Convention on the Rights of the Child, and the Global Action Plan for Pneumonia and Diarrhea (GAPPD) to monitor and compare indicators, publishing on performance for anywhere from 18 (Goalkeepers) up to 56 measures (Scorecard on State Health System Performance).

Seven tools used a global frame of reference; four analyzed a subset of countries, such as countries in the Americas; three addressed health within the United States; and two focused on individual U.S. states (Vermont and Washington). Four resources were called scorecards, nine were dashboards, and two did not use either term. The differences between web-based scorecards and dashboards were nonobvious, in that both types of platforms used color styling to characterize performance and visualized data updated quarterly, annually, and as needed, but not in “real time”; however, the ALMA and NCD scorecards typified the scorecard genre with color-coded tables, which could be quickly scanned by decision makers. Aside from COVID-19, most platforms focused on child health-related metrics, from core priorities like immunization and malaria control to economic measures like the proportion of children in poverty.

To orient readers, the scorecards used colors to signal performance, typically with reddish shades signaling poorer measures. Most (10/15) platforms displayed tables, about half (9/15) used charts, and eight showed data on maps. About half of the platforms enabled data downloading into PDF formats, for instance, to view annual reports, as with the Goalkeepers and CommonWealth tools.

Dashboards and scorecards often described how they engaged core stakeholders, for instance in pages explaining their purpose, funding sources, and impact. Some organized events to engage policymakers, such as ALMA, Countdown to 2030, Goalkeepers, the Congressional District Health Dashboard, and the JHU dashboard. These platforms featured examples of how policy engagement with visualized data translated to improved health measures, such as with an ALMA malaria scorecard:

“Officials saw the proportion was low… this indicator was red showing it was not on track…” Officials took action with “coverage increasing from 42% to 83% in the province.”

Other platforms referred to numerous media citations. The State-by-State Look at Coronavirus in Prisons by the Marshall Project described how articles referring to visualized data from the platform influenced elected officials to take actio“:

“Members of Congress immediately started citing our figures in their own demands for a response.”

As several vaccine scorecards described in the peer-reviewed literature were not intended to be updated [28,29], we sought to review public platforms that were updated periodically with the availability of new data. Ten of 15 tools were up-to-date as of May 2023, as in showing data from the current period (monthly or annual), although several COVID-19 dashboards, such as the JHU platform, were no longer updated after the CDC/WHO determined the pandemic to have ended.

### 3.4. Merged Analysis

To better understand whether the participants’ views aligned with global trends in the visual analytics movement, we mapped views across six themes and compared them with dashboards and scorecards examined in the document review (Table 2). *IA2030 Intended Use* and *Visualization Gaps* were omitted from this table because of their specificity to the IA2030 scorecard.

We found some concurrence across five themes and mixed findings across four themes; there were similarities and differences within *data*, *advocacy*, and *understandability*. Within *Tools*, we noted many dashboards were created to visualize COVID-19, a theme discussed by nearly all participants. Regarding *Advocacy*, many platforms aim to highlight service gaps and drive actionable decision making, with the majority intending to reach policymakers. Furthermore, platforms are mainly set up to enable comparisons between geographies. The potential for constructive comparisons was identified by country-level participants and some of the regional participants. Finally, nearly all the tools used a reddish color to signal poor performance, which the scorecard does as well. However, the scorecard does not use green, which many platforms used to signal good performance; the lack of green color for this purpose was noted by several key informants.

The theme with the most mixed findings was *Health System Challenges*, with participants mainly discussing upstream management and human resourcing limitations, whereas platforms mainly focused on service delivery. There were mixed findings within *Data*. Whereas country-level participants said that reviewing 18 indicators on the scorecard’s Overview page was overly complex, some tools had more than twice that number of indicators; further, one-third of the tools had not been regularly updated, risking irrelevancy, which the key informants discussed as a pitfall. While the scorecard’s stated mission to increase accountability, commitment, and resources is similar to that of the other tools, its initiatives did not include policymaker-focused events at the time of the research; this contrasted with the one-third of platforms reviewed, which were tied up with initiatives organizing designated events to display the data to policymakers.

## 4. Discussion

Many public platforms with data visualizations have been launched as accountability mechanisms serving a variety of audiences. Leveraging a strategy that originates in the balanced scorecard tool developed for optimizing commercial performance, these platforms are used to increase stakeholders’ awareness of areas of progress and deficiencies. We sought to characterize the context for implementation of the Immunization Agenda 2030 (IA2030) scorecard with a sample of intended users using components of a popular metaframework, the Consolidated Framework for Implementation Research. We further sought to understand the context by examining the characteristics of 15 other publicly available visual analytics platforms developed for health performance tracking and accountability. Finally, we aimed to assess whether stakeholder expectations for the IA2030 scorecard reflect an understanding of how other platforms are developed and used in many contexts. While the number of platforms has proliferated in recent years, this analysis appears to be one of the first in the peer-reviewed literature to describe a systematic effort engaging globally diverse intended users to review a publicly available population health platform for its usability and appropriateness. Furthermore, this was the first assessment of the IA2030 scorecard.

We set out to describe the information needs of program officers seeking to monitor and communicate about immunization performance. The pandemic, discussed by participants as being prominent in the external context (outer setting) for IA2030 scorecard implementation, spurred the development of manifold specialized digital platforms used by immunization managers for population health data monitoring and agenda setting. Platforms highlighted service gaps, seeking to enable actionable decision making by policymakers, which interview participants also felt was a key use case for the IA2030 scorecard. The tools often compared geographies, such as U.S. counties, to spur positive pressure for performance improvements.

There were differences between the interview perspectives on digital tools and what we found in our document review. Other than the platforms focusing on COVID-19, most tools highlighted child health service outcomes, whereas participants’ leading health system concerns were management capacity and governance. Furthermore, at least one-third of the tools we reviewed were not up-to-date, risking irrelevancy, which participants discussed as a pitfall of establishing public data platforms. However, participants did not always distinguish between provisional data—such as that shared through ubiquitous COVID-19 data dashboards—and official data aggregated by WHO, such as annually compiled WHO and UNICEF estimates of national immunization coverage (WUENIC) coverage estimates [30]. Integrating provisional and official data into the scorecard could bolster relevancy for policy action. Yet this potentially increases the political sensitivity, flagged as a risk by some participants.

In our review, platforms often described their mission as seeking to increase accountability and foster data-driven decisions, with one-third explicitly describing events organized to highlight the platform’s data to policymakers often concerned with pandemic recovery. This is aligned with the function of the balanced scorecard in healthcare settings, which effectively focuses the attention of managers on the achievement of core objectives [31]. However, such actors must be explicitly engaged. Indeed, a review of pandemic-focused dashboards concluded that metrics “will be of little value unless they are disseminated in the form of timely, actionable information that rapidly reaches… actors… in a position to support the larger public health goals” [17]. Other platforms, like the Marshall Project’s State-by-State Look at Coronavirus in Prisons listed hundreds of media articles citing the data and related discussions in the U.S. Congress [32,33]. Advocacy was the third-most common theme discussed in interviews, with participants similarly emphasizing the importance of using the scorecard for briefing leaders. The scorecard’s features, such as the ability to compare between countries, were cited as useful for spurring positive pressure among leaders for corrective actions. Another qualitative study exploring the design of a scorecard for global diarrhea control similarly found participants recommended facilitating country comparisons to enhance accountability [34]. Fostering healthy competition through benchmarking performance has been a staple of balanced scorecard approaches in multiple contexts [35].

Participants identified opportunities to enhance the scorecard design as well as the initiative’s engagement with its targeted users. Country-level stakeholders noted opportunities to simplify the visualizations, for instance in the overview dashboard and country page. Dynamic visualizations differentiate the scorecard from reports issued to highlight immunization performance gaps. Visual analytics platforms leverage “human cognition in processing visual representations,” and they enhance learning by enabling decision makers to interact with data [36]. In a U.S. hospital setting, making “easy-to-read” data charts for health leadership has similarly motivated strategic planning [11]. To align with the participants’ expectations and the 15 tools reviewed, the IA2030 Scorecard could utilize simplified visualizations to better engage with a policy audience that can allocate resources, guide planning, and ensure accountability. Additionally, participants speaking from a WHO regional perspective noted the need for adaptation at the regional level to be congruent with diverse regional policy agendas, as shown in Box 1. Other platforms reviewed, such as Countdown to 2030, have taken similar approaches to ensure that country profiles enable cross-regional comparisons but are also adapted with some additional customized pages to make sure they are relevant to regional and country planning purposes.

This study of implementation context was carried out principally to inform the scorecard’s design and strategy to encourage adoption. Participants’ insights, such as the need for data specificity and data annotation on the overview dashboard, have informed the current design and future priorities. While we reached saturation with our interview participants, due to the need for a rapid response from a variety of regional and country-level specialists, we could not compare thematic findings between regions. We also recruited and interviewed English-speaking participants, and thus perhaps over-sampled Anglophone regions, although many immunization officers working at the national and regional level have professional competency in English. Also limiting our geographic representativeness, we were unable to recruit participants from the Western Pacific Region. Furthermore, although we encouraged participants to share criticism on the scorecard’s design, noting that the interviewers had not personally designed the tool and highlighting substantial suggestions and negative feedback from many participants, some respondents likely tempered their feedback due to a social desirability bias.

## 5. Conclusions

The Immunization Agenda 2030 scorecard is among a range of new visual analytics platforms aiming to foster greater stakeholder engagement with population health data. Like many such tools, it aims to increase shared accountability and performance. Immunization stakeholders consulted in the study felt the IA2030 scorecard can empower regions and countries to enhance their immunization systems by providing a tool for assessment, benchmarking, and decision making. Participants felt the IA2030 scorecard would serve as a resource in the global effort to improve immunization coverage and equity. Globally diverse immunization officials interviewed felt the scorecard’s design was compatible with their needs but could be simplified in places to ensure rapid dissemination of immunization systems performance, particularly for actors at the country level. The rapid consultation and document review provided immediate feedback for the scorecard’s implementation team to identify opportunities to make design and implementation choices that would better focus attention on performance deficits and strengths. With the recommended enhancements, the scorecard has the potential to ensure greater accountability at all levels to optimize the potential of global immunization services.

## Figures and Tables

**Table 1:** Interview participant characteristics.

Interview #	Level	Gender	Job Description	WHO Region
1	Regional	Female	Program Manager	AMR
2	Subnational	Male	M&E	AMR
3	National	Male	Technical Advisor	AMR
4	Regional	Female	Program Manager	EUR
5	National	Male	Program Manager	AFR
6	National	Male	Program Manager	EMR
7	Regional	Male	M&E	EMR
8	National	Male	Program Manager	EMR
9	Regional	Male	Program Manager	AFR
10	National	Male	M&E	AFR
11	Subnational	Male	M&E	EMR
12	Regional	Female	Program Manager	AFR
13	National	Male	Technical Advisor	AFR
14	Regional	Male	Program Manager	AMR
15	Regional	Male	Program Manager	SEAR

**Table 2:** Merged thematic analysis.

Theme	Outcome + Concurrence - Mixed Findings	What Did the Platforms Show?	What Did the Key Informant Participants Say?
Outer Setting Themes			
Tools	+	Large number of COVID-19 dashboards, with six reviewed.	Increased use of visual dashboards during pandemic
Health system challenges and COVID disruptions	-	Visualized routine child, quantitative health indicators	Concerned about management capacity, financial challenge, governance limitations, health worker exhaustion
Inner Setting Themes			
Data	+	U.S.-focused platforms visualized data by state and county-level.	Sought to use scorecard for district, regional and community-level planning and advocacy
	-	Large numbers of indicators were displayed by all platforms One-third of platforms were not updated in 2023.	Country-level participants found “Dashboard,” with 18 indicators, overly complex. Platforms should be regularly updated to avoid irrelevance.
Advocacy and Priorities	+	Platforms are described as helping identify service gaps, drive actionable decision making.	Envisioned using scorecard for rapid policy briefing, stakeholder engagement, support data-driven decisions
	-	One-third of sample platforms had related events to capture attention of policy makers.	While participants said policy-focused events would help foster change, IA2030 scorecard does not describe such events.
Comparison	+	Most platforms explicitly set up for comparison between countries or other geographic entities	Aimed to compare country performance to peers to create “positive pressure”
Product Characteristics			
Understand-ability	+ -	Eight of the 15 tools signal their intention to be accessible and “easy to use.” Dashboards tended to be more complex.	Regional level users felt scorecard is easy to use. Country-level users felt the overview dashboard, with 18 indicators, should be simpler.
	-	Platforms used reddish color to signal poor performance and green for good performance.	Scorecard uses reddish and yellow color to signal poor performance. Does not use green.

## Data Availability

Due to privacy considerations inherent in key informant interviews, data are not publicly available for this study.

## Appendix A. Template for Framework Chart

- Overarching Perspective: Summary
- Illustrative Quotes (1–3):

**Category**	**Code**	**Definition**	**Quote**	**Summary**
Instructions				
Copied from codebook			Include the best 2–3 quotes with this code in the transcript. Remove “uh” and other filler words.	Condense informants’ comments on this code across all coded quotes in transcript. Note where codes are overlapping.
Outer Setting	Health System Challenges	Barriers to sustaining and improving routine immunization performance, such as financial and personnel management.		
	Tools	Discussion of data visualization or management tools to monitor immunization systems at the country or regional level.		
	COVID-19 Disruptions	Any mention of COVID-19, particularly the financial and human resources related to pandemic response, COVID-19 vaccine coverage, and disruptions to immunizations		
Inner Setting	Advocacy	Potential use of scorecard in making advocacy presentations or other high-level communications to policy makers or other influencers. Includes use of scorecard for decision making and implementation. Includes references to the certain level it will be used at (regional, country-level, subnational) and stakeholders.		
	Data	Discusses units used to display data, such as number of outbreaks, number of people, number of deaths. Refers to the need for standardization of units or clear indication of what units a visualization uses. Could also reference where the data should come from (i.e., regional or country level) or a lack of data (e.g., missing data in general or on scorecard website).		
	Comparison	Discusses either the need for the dashboard to provide suitable means for comparison across indicators and regions or country-level comparisons		
	IA2030 Intended Use	Refers to adaptation/tailoring of IA2030 objectives and indicators for country or region. Not specific to scorecard (can be double coded).		
	Priorities	Refers to current or new changes in prioritization in immunization goals, including decision making based on the use of data. Not necessarily specific to IA2030 framework/scorecard (can be double coded).		
Product Characteristics	Visualization Gaps	Features missing from scorecard		
	Understandability	Ease or difficulties in interpretation/understandability of the dashboard. This could refer to the language, the visualization, color scheme, or the meaning of on track-off track. Also includes ease or difficulties in interpreting the overview dashboard.		

## Appendix B. Platforms Reviewed

**Name of Platform**	**Website Address**
State of Vermont Immunization & Infectious Disease Scorecard	https://embed.clearimpact.com/Scorecard/Embed/600 (accessed on 29 May 2023)
The ALMA Scorecard for Accountability and Action	https://scorecardhub.org/guides-and-toolkits/country-level-scorecard-tool-introduction/ (accessed on 29 May 2023)
Countdown to 2030 Dashboards	https://data.unicef.org/countdown-2030/ (accessed on 29 May 2023)
NCD Scorecard	Archive image: https://www.c3health.org/blog/global-ncd-scorecard/ (accessed on 29 May 2023)
NTD ConnectOR	https://www.cor-ntd.org/research-outcomes/dashboard (accessed on 29 May 2023)
Goalkeepers	https://www.gatesfoundation.org/goalkeepers/ (accessed on 29 May 2023)
VIEW-hub	https://view-hub.org/ (accessed on 29 May 2023)
UNICEF Immunization coverage estimates dashboard	https://data.unicef.org/resources/immunization-coverage-estimates-data-visualization/ (accessed on 29 May 2023)
Congressional District Health Dashboard	https://www.congressionaldistricthealthdashboard.org (accessed on 29 May 2023)
The Marshall Project: COVID Cases in Prisons	https://www.themarshallproject.org/coronavirus (accessed on 29 May 2023)
COVID-19 data of the Region of the Americas	https://www.paho.org/en/topics/coronavirus-infections/coronavirus-disease-covid-19-pandemic (accessed on 29 May 2023)
Washington State COVID-19 Data Dashboard	https://doh.wa.gov/emergencies/covid-19/data-dashboard#technical (accessed on 29 May 2023)
Johns Hopkins Coronavirus Resource Center	https://coronavirus.jhu.edu/ (accessed on 29 May 2023)
2022 Scorecard on State Health System Performance	https://www.commonwealthfund.org/publications/scorecard/2022/jun/2022-scorecard-state-health-system-performance (accessed on 29 May 2023)
WHO Coronavirus (COVID-19) Dashboard	https://covid19.who.int/ (accessed on 29 May 2023)
